# Modelling the dynamics of a large rock landslide in the Dolomites (eastern Italian Alps) using multi-temporal DEMs

**DOI:** 10.7717/peerj.5903

**Published:** 2018-11-08

**Authors:** Ricarda Gatter, Marco Cavalli, Stefano Crema, Giulia Bossi

**Affiliations:** 1 MARUM—Center for Marine Environmental Sciences, University of Bremen, Bremen, Germany; 2 CNR-IRPI—National Research Council, Research Institute for Geo-Hydrological Protection, Padova, Italy

**Keywords:** Landslide, Modelling, Digital Elevation Model, DEM of Difference, Back analysis, Seismic data, UNESCO, Dolomites UNESCO World Heritage, 2021 Alpine Skiing World Championship, Geomorphometry

## Abstract

Latest advances in topographic data acquisition techniques have greatly enhanced the possibility to analyse landscapes in order to understand the processes that shaped them. High-resolution Digital Elevation Models (DEMs), such as LiDAR-derived ones, provide detailed topographic information. In particular, if multi-temporal DEMs are available, it is possible to carry out a detailed geomorphic change detection analysis. This analysis may provide information about the dynamics of large landslides and may thus, be useful for landslide risk assessments. However, LiDAR-derived DEMs are mostly available only as post-event surveys. The technique is relatively recent, and local or national authorities only started widespread surveys in the last decade. Therefore, it is of a certain interest to analyse the effectiveness of DEMs derived from technical cartography to produce reliable volumetric estimates related to large landslides. This study evaluates the use of a multi-source DEM of Difference (DoD) analysis for the investigation of a large landslide –Le Laste–, which occurred on November 12, 2014 on Mount Antelao (eastern Italian Alps). The landslide initiated as a 365,000 m^3^ rockslide close to the summit of the mountain and transformed into a debris avalanche during its runout. The comparison of pre- and post-event DEMs allowed for the identification and quantification of erosion and deposition areas, and for the estimation of landslide volume. A sound back-analysis of the landslide with the 3D numerical model DAN3D was based on this comparison and on seismic records of the event. These seismic records proved to be remarkably useful, as they allowed for the calibration of the simulated landslide velocity. This ensured the reliability of the model notwithstanding the topographic datasets, intrinsic uncertainties. We found that using a pre-event DEM derived from technical cartography tends to slightly overestimate the volume with respect to the use of the more accurate LiDAR-derived DEM. In recent years, the landslide risk around Mt. Antelao has been increasing alongside the ever-growing population and human activities in the area. Sediment accumulations produced by the Le Laste landslide significantly amplified the debris flow hazard by providing new sediment sources. Therefore, it is crucial to delineate the distribution of this material to enable an adequate debris flow hazard assessment. The material properties derived from the back-analysis of the Le Laste landslide can be used to simulate the runout of possible future events, and to generate reliable hazard zone maps, which are necessary for effective risk mitigation.

## Introduction

Landslides occur over a wide range of spatial and temporal scales in mountainous areas all over the world and pose a substantial threat to people, property, and infrastructure (e.g.,  Palmquist & Bible, 1980; Corominas, 1996; Hungr, Corominas & Eberhardt, 2005). In Italy, about three-quarters of the territory (Canuti et al., 2004) are covered by mountains, which are subject to mass movements of different types and sizes. The average death toll rate related to landslides reached 59 victims per year in the last century (Guzzetti, 2000) and the Italian National Research Council, Institute for Geo-Hydrological Protection (CNR-IRPI) estimated that the total cost of direct damage ranges between 1 and 2 billion Euros per year (Canuti et al., 2004). This highlights the importance of landslide risk assessment and management, especially in densely populated regions, in order to prevent people and human activity from being exposed to such hazards (Guzzetti, 2000).

The prediction of landslide volume and runout is a key requirement for the development of hazard zone maps. The determination of dynamic parameters, such as landslide velocity and depth, is crucial for the implementation of protection measures (Crosta, Chen & Frattini, 2006). However, since landslide processes involve numerous variables (Corominas, 1996), risk assessment can be very complicated. Moreover, the understanding of these processes strongly depends on the availability and the quality of topographic data (Brasington, Rumsby & McVey, 2000).

Advances in survey technology, digital terrain modelling, and GIS have increased the capabilities of landslide detection and monitoring (e.g.,  Brasington, Rumsby & McVey, 2000; Tarolli, Arrowsmith & Vivoni, 2009). Mass movements can be identified by visually interpreting derivatives of high-resolution Digital Elevation Models (DEMs; e.g.,  McKean & Roering, 2004) and by means of direct comparison of multi-temporal DEMs in the form of DEM of Difference (DoD) maps. DoD maps have been successfully applied to quantitatively assess morphological changes caused by earthflows (Corsini et al., 2009; DeLong et al., 2012; Daehne & Corsini, 2013), debris flows (Scheidl, Rickenmann & Chiari, 2008; Bull et al., 2010; Theule et al., 2012; Blasone et al., 2014; Cavalli et al., 2017), and other types of landslides (Baldo et al., 2009; Burns et al., 2010; Tseng et al., 2013; Chen et al., 2014). In addition, they can be used to calibrate numerical models, which rely on the back-analysis of prototype events (Bossi et al., 2015; Gregoretti, Degetto & Boreggio, 2016). Jaboyedoff et al. (2012) provided a review on the use of multi-temporal LiDAR-derived DEMs for monitoring mass wasting processes.

Numerical models have been widely applied to investigate geomorphological processes (e.g.,  Hungr, Corominas & Eberhardt, 2005; Rickenmann, 2005). They can simulate different hazard scenarios, illustrating potential landslide runout and material distribution. With this information, risk assessments can be carried out and hazard maps can be established to reduce the associated risk (Crosta, Chen & Frattini, 2006). However, apart from the input rheological parameter values, detailed topographic information is needed to model the behaviour of landslides accurately (Bossi et al., 2015; Sauthier et al., 2015; Phillips et al., 2017). Although accuracy and resolution of DEMs have improved through the application of laser scanning techniques such as LiDAR (Light Detection and Ranging), multi-temporal data is not always obtainable. Usually, surveys are only undertaken after landslide occurence and not on a regular basis. Therefore, combining datasets from different DEM sources could be the only solution to investigate landslide dynamics, and must be carried out by carefully assessing the quality and accuracy of the input DEMs and by taking into account error propagation (uncertainty) in the final DoD.

In this study, the effectiveness and limitations of multi-temporal DEM analysis, carried out considering different sources and at different resolutions, were evaluated. The focus of the work is on the Le Laste landslide that occurred on November 12, 2014, on Mount Antelao in the eastern Italian Alps. The research aimed to reconstruct the dynamics and behaviour of the landslide, and to evaluate the remaining hazard potential for the region around Mt. Antelao by combining DoD analysis and 3D modelling.

The selected landslide acted as a suitable example due to the availability of pre- and post-event DEMs, as well as seismic records from different seismographs and rainfall data.

Some authors have discussed the potential of seismograph stations recording the ground vibration of mass movements to detect and localise landslides in uninhabited areas (Jeffreys, 1923; Norris, 1994; Weichert, Horner & Evans, 1994; Suriñach et al., 2005; Lin et al., 2010). In addition, since direct observations of landslides are often unavailable, seismic data can give valuable details on the kinematics and dynamics of long runout landslides by providing information about the velocity and intensity of the phenomenon (e.g.,  Guthrie et al., 2012; Allstadt, 2013). This information, in turn, can be used to calibrate rheological parameters and to validate numerical models (Pirulli, 2009; Schneider et al., 2010; Moretti et al., 2012).

DoD analysis from technical cartography maps and LiDAR data enabled the delineation of the landslide boundaries, the illustration of erosion and deposition patterns, and the estimation of the landslide volume. The results of this analysis allowed the calibration of a 3D dynamic model (DAN3D; McDougall & Hungr, 2004) by matching the simulated travel distance, and the extent and depth of the deposit to the real event. Additionally, seismic records provided information on the initial landslide velocity (Manconi et al., 2016), which could be used during the calibration of the numerical model.

Another objective of this work was to test the potential of widely available data to characterise large landslides in remote areas. LiDAR-derived DEMs are expensive and not always available, especially for pre-event conditions. Therefore, it was considered important to evaluate the usability of DEMs derived from technical cartography to produce reliable volumetric estimates related to large landslides. For this purpose, the results derived from two different DoD analyses were compared. The first was performed on pre- and post-event LiDAR-derived DEMs, and the second used a technical cartography pre-event DEM and a LiDAR post-event DEM as input files.

## Study Area

### Geographic and geological setting

**Figure 1:**
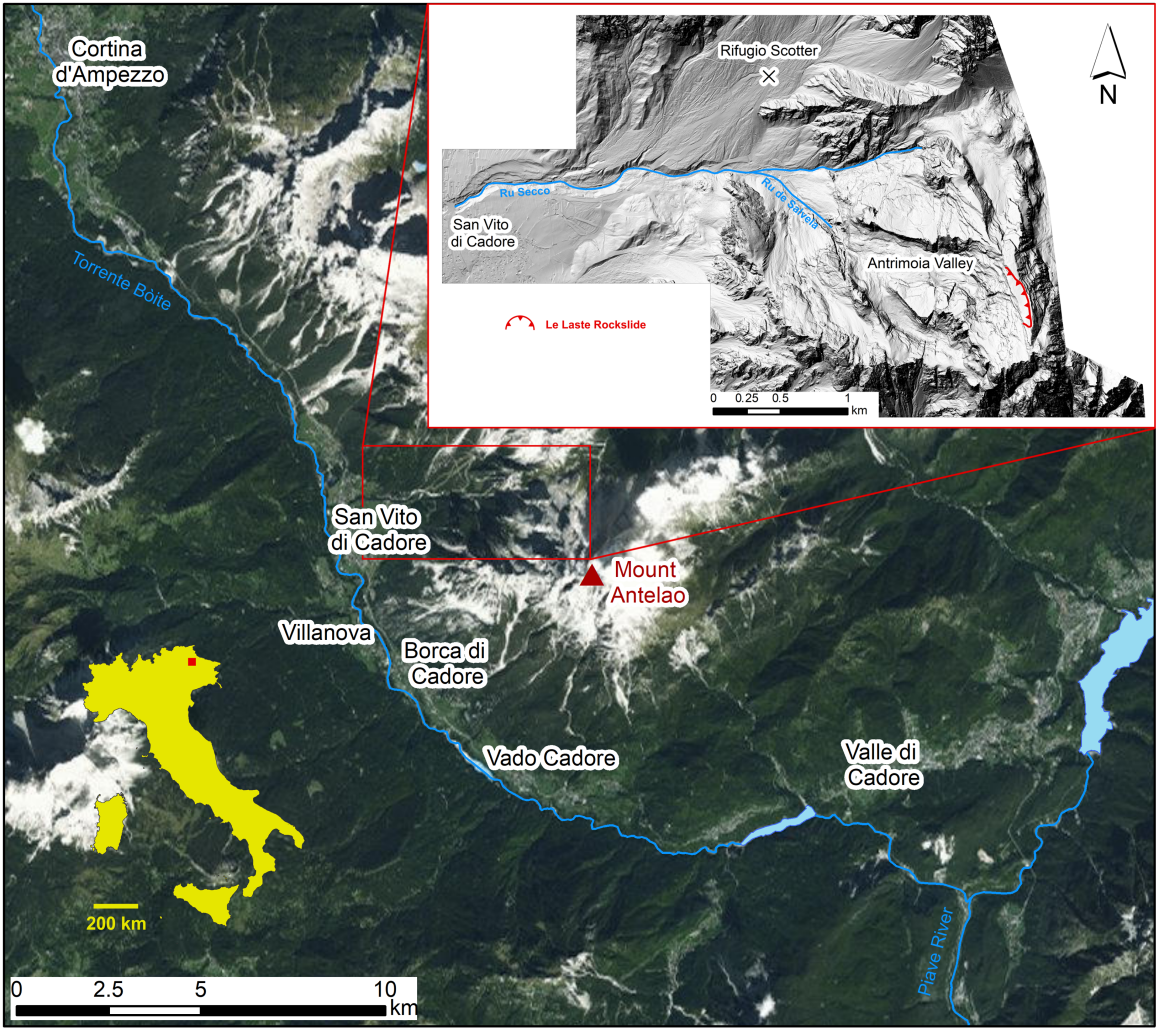
Location map of the study area.

Mount Antelao (3,264 m a.s.l.) is the second highest mountain in the Dolomites, a UNESCO world heritage site in northeastern Italy (Fig. 1). It lies southeast of the municipality of Cortina d’Ampezzo and northeast of the Boite valley that is crossed by the Torrente Boite in N-S direction, a right tributary of the Piave River. On the southern side of the Antelao complex lies the very steep Valón dell’Antelao. The Ru Secco valley that runs through the municipality of San Vito di Cadore, borders Mt. Antelao in the North and a massive rocky bank that is known as “Le Laste” defines the western aspect (Baglioni & De Marco, 2015). Two longer valleys descend on the northeastern side and are crossed by streams of glacial origin. The mountain group conceals two small glaciers, the “Superiore” and the “Inferiore”. Both glaciers are located at the head of U-shaped valleys that converge in the Piave valley (Sudiro, 2004).

 The very steep, rocky walls of the Antelao massif are formed primarily of calcareous-dolomitic rocks, which belong to the “Dolomia Principale” formation and the overlying “Calcari Grigi” formation. The maximum thickness (about 1,000 m) of the Dolomia Principale can be found north of the study area, while only the upper part of the formation appears on the surface within the research area. The Calcari Grigi reach a thickness of about 500 m.

The stratigraphic succession outcropping in the research area covers a time period from Upper Triassic to Lower Jurassic and is described in the geological map produced by Neri et al. (2007) and in Bosellini (1996).

The Dolomia Principale formation (Carnian–Rhaetian) consists predominantly of clear or white to grey, well-stratified dolomite, in which stromatolitic and massive lithozones can be found. The formation can be divided into three main units. The lower and upper units are well-stratified and formed by the cyclic succession of peritidal dolomite. The middle unit is less distinguishable stratified and probably bioturbated. The Calcari Grigi formation (Rhaetian –Lower Lias) consists of well-bedded light grey micritic limestone and whitish bioclastic and oolitic limestone. The formation is organised in peritidal cycles and quite homogeneous, with only slight differences between the upper and lower part.

The Boite valley is characterised by intense human activity. It constitutes one of the major communication routes between Italy and Austria, and represents one of the most touristic areas in the Alpine Region. Moreover, touristic development is bound to increase, since the Alpine Skiing World Championship in 2021 will be held in Cortina d’Ampezzo.

Various types of mass movements often affect the region around Mt. Antelao (Panizza et al., 1996; Pasuto & Soldati, 1999; Pasuto & Soldati, 2004; Gregoretti & Dalla Fontana, 2008). The geomorphological hazard results from three main factors. (1) Geotechnical properties and structural conditions of the area that allowed the formation of steep slopes due to the erosion of weaker layers along the contact with more resistant rocks (Soldati & Pasuto, 1991). (2) The tectonic activity, which caused intense jointing in correspondence with the principal faults in the Dolomites, thus forming discontinuities, which become potential sliding surfaces (Carton & Soldati, 1993; Panizza et al., 1996). (3) The effects of intense glacialism that dominated the region during the Würmian period caused rock deformations in correspondence with structural discontinuity surfaces (Panizza et al., 1996; Borgatti & Soldati, 2010).

The frequency and magnitude of gravitational phenomena were especially high in the last post-glacial period, when slopes were no longer sustained by ice masses (Carton & Soldati, 1993; Borgatti & Soldati, 2010). The repeated gravitational movements included many debris flows, but rock and debris avalanches also caused severe destruction and a number of casualties. Among those, a landslide that occurred on May 2, 1730 on Mt. Antelao reached the municipality of San Vito di Cadore, leading to 51 deaths (Guzzetti, Stark & Salvati, 2005) and rock avalanches on April 21, 1814, and February 17, 1925, led to death toll estimates of over 300 deaths each (Canuti et al., 2004). Other important historical events occurred on January 25, 1348, in June and July 1737, on July 27, 1868 (Bacchini & Zannoni, 2003; Pampanini, cited in Sauro, 2000), on August 7, 1996 (Panizza et al., 1998) and in July 2009 (Deganutti & Tecca, 2013).

### Le Laste Landslide

**Figure 2:**
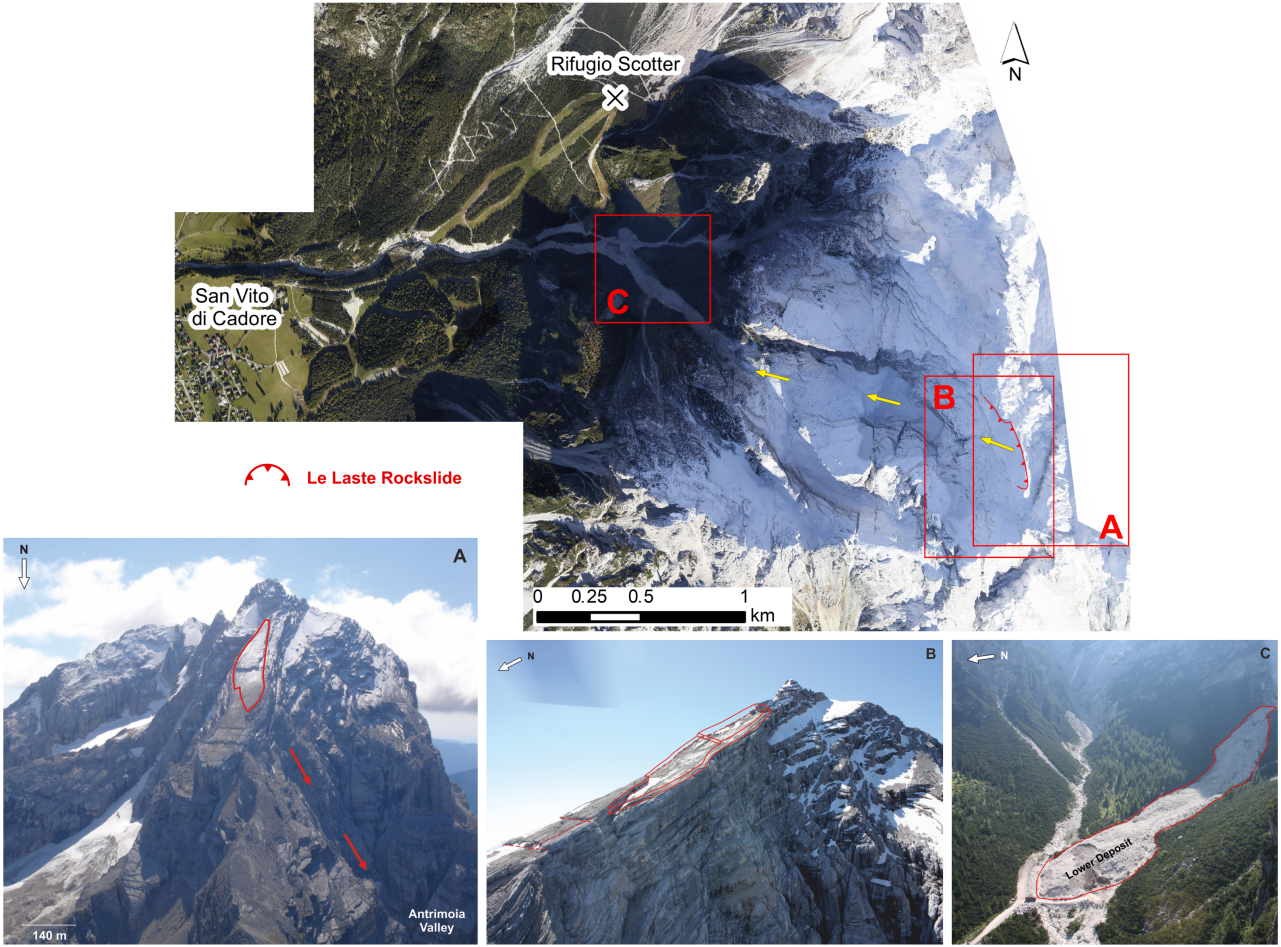
Orthophoto of the landslide area. Yellow arrows illustrate the main pathway of the landslide. (A) Failure zone before the event. (B) Failure zone after the rockslide detached from Mt. Antelao. (C)“Lower” deposit damming Ru Secco after the debris flow in 2015 (Photo credit: Palmiro De Marco).

On November 12, 2014 a rockslide, known as “Le Laste”, detached from the summit of Mt. Antelao. It initiated close to the mountaintop (3,264 m a.s.l.), in an area characterised by a strong stratigraphic layering of the dolomite. During its runout of more than 2 km, the rockslide evolved into a debris avalanche (as defined by Varnes, 1978) that almost reached the confluence of the Ru de Salvela and the Ru Secco, roughly 200 m from the access road to the Rifugio Scotter (Baglioni & De Marco, 2015; Fig. 2). Baglioni & De Marco (2015) provided observations of the Le Laste landslide based on an aerial survey, which was conducted on July 1, 2015, and an estimation of the initial failure volume of about 300,000 m^3^.

**Figure 3:**
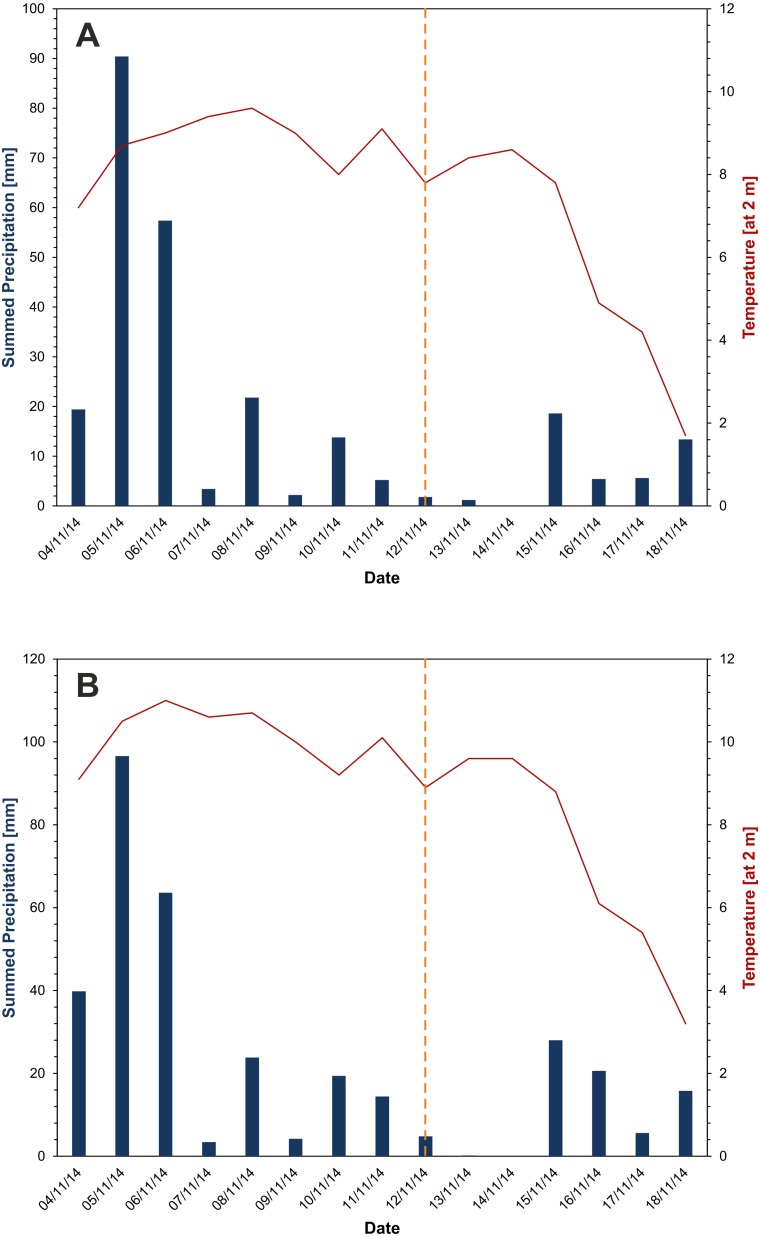
Precipitation and Temperature before and after the event. Precipitation (blue) and average temperature (red) data before and after the Le Laste landslide from (A) Villanove (Borca di Cadore) and (B) Valle di Cadore. The date of the landslide is illustrated in orange.

The failure zone is located between approximately 2,800 and 3,100 m a.s.l. The mobilised block slid according to the dip direction of the bank layers, before falling about 600 m down onto the Antrimoia Valley. The wall that links the failure zone and the Antrimoia Valley consists of a system of subvertical discontinuities, which are oriented approximately NO-SE and allowed the whole rockslide to fall towards the valley. The fall over an elevation drop of 600 m resulted in the complete break-up of the rockslide and subsequent debris avalanche runout behaviour, with a high degree of deposit gradation. During runout, parts of the material remained in the Antrimoia Valley and formed the “Upper Deposit”. The rest of the landslide moved down another step and accumulated along the Ru de Salvela, forming the “Lower Deposit”. Due to rainfall between 15 and 18 November 2014 that was recorded by the meteorological stations in Villanova and Valle di Cadore (by A.R.P.A.V. Centro Meteorologico di Teolo; Fig. 3), more material was eroded from the mountain site and the accumulation front advanced further. Nevertheless, it has to be noted that the rainfall during that time did not deviate from the monthly average and that the temperatures were mild for the season.

## Methods

### Datasets and data preparation

**Figure 4:**
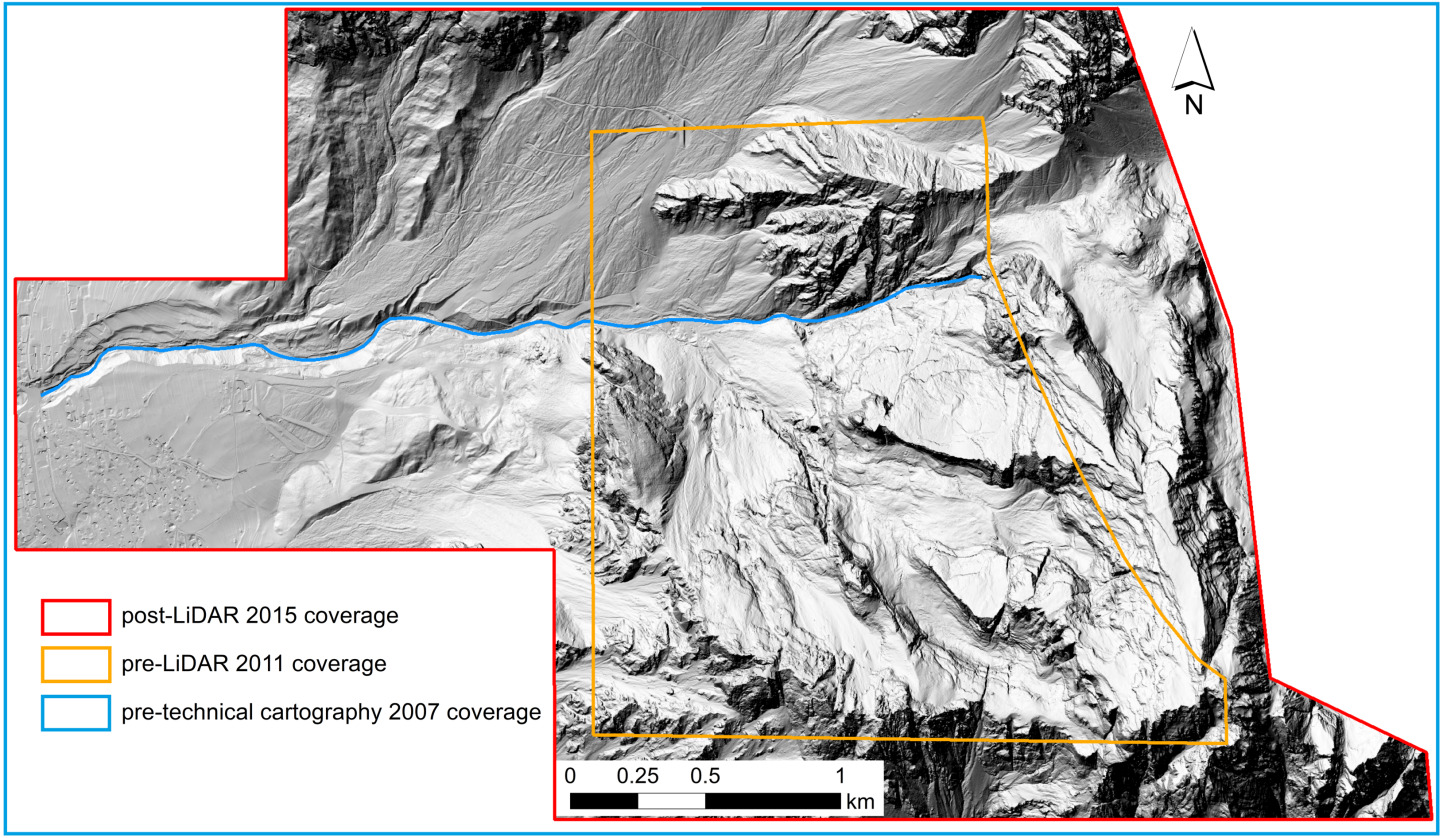
Outlines of the dataset coverages.

Three topographic datasets were available for the study area and were used to identify geomorphic changes related to the 2014 Le Laste landslide (Fig. 4). For the first dataset, a 1 m resolution DEM representing the pre-event topography was elaborated from LiDAR data collected by the Province of Belluno in November 2011. Since the 2011 LiDAR point cloud did not cover the whole studied area, it was integrated with technical cartography data from 2007, which covered the missing area. The two point clouds were aligned to one another and merged into a single 1 m resolution pre-event DEM.

In addition, a second pre-event DEM with a resolution of 5 m was provided by the Regione Veneto cartographic service and it is based on 2007 technical cartography data (scale 1:5,000, 5 m contour interval). The additional pre-event DEM was used to investigate the effectiveness of largely available data covering wide areas (e.g., the ones derived from technical maps) to provide reliable results in multi-temporal DEM analyses.

It is worth highlighting that both pre-event DEMs that are used in this study date back to years rather distant in time from the event (i.e., 2007 and 2011). Thus, a DoD derived from them is affected by an uncertainty related to the different hydro-geomorphic processes that occurred in the considered time window.

The third dataset indicated the post-event conditions. The DEM was derived from LiDAR data collected by the MATTM (Ministero dell’Ambiente e della Tutela del Territorio del Mare) in the Antelao region on October 21 and 23, 2015. The available data consisted of 13 tiles that were already interpolated with a triangulation algorithm and then converted into a 1 m resolution grid using linear interpolation. These files were then merged into a single DEM.

The three DEMs were used to help to identify the landslide scarp, to calculate the initial failure volume, and to quantify the topographic changes within the landslide-affected area by computing a DEM of difference (DoD) map.

The DoD results are highly sensitive to the alignment of the point clouds (Lallias-Tacon, Liébault & Piégay, 2014) and to the quality of the DEMs used in the analysis (Brasington, Rumsby & McVey, 2000). Therefore, in order to achieve a higher spatial coherence between the pre- and post-event DEMs, the pre-event point clouds were realigned according to the post-event point cloud with Cloud Compare (http://www.danielgm.net/cc/), a 3D point cloud and mesh processing software. For the LiDAR post-event and cartographic pre-event analysis, the cartographic pre-event point cloud was converted into a 5 m resolution grid and the original post-event LiDAR DEM was resampled to a 5 m resolution. Special attention was put onto grid concurrency, which is needed in order to produce an accurate DoD map. The original 5 m resolution of the cartographic DEM was maintained because the point cloud density could not be increased adequately over the whole study area to obtain a finer resolution as suggested by Degetto, Gregoretti & Bernard (2015). Furthermore, increasing the cartographic DEM resolution from 5 m to 1 m through a resampling operation would have produced a larger error in the analysis than resampling the LiDAR DEM from 1 m to 5 m.

For the analysis of the LiDAR post-event and integrated LiDAR and technical cartography pre-event, the highest possible resolution of the input DEMs (i.e., 1 m) was used. This operation might introduce some uncertainty. However, the technical maps were only used in the vicinity of the detachment area. The detachment area is characterised by linear and homogeneous slope morphologies, and the rendering accuracy for such areas can be considered high. The absence of complex morphologies in the detachment area represents a positive prerequisite for a reliable resampling operation.

### DEM of Difference (DoD) computation

A key advantage of DEM differencing relies on its potential to identify the distribution and mass balance of topographic changes by directly comparing multi-temporal topographic surveys (Brasington, Rumsby & McVey, 2000). To determine the extent of the area affected by the Le Laste landslide, the integrated LiDAR and technical cartography pre-event DEM and post-event LiDAR DEM were subtracted. This allowed for a preliminary comparison between the DEMs by computing the magnitude and direction (erosion or deposition) of change on a cell-by-cell basis. Using a grid-overlay function, however, does not take into consideration uncertainties and errors in the analysis (Brasington, Rumsby & McVey, 2000). In particular, potential errors linked to the vertical and horizontal precision and accuracy of the LiDAR data (Cavalli & Tarolli, 2011), the effect of filtering processes applied to remove vegetation and man-made features from the raw data set, and the effect of snow cover (Deems, Painter & Finnegan, 2013) were not considered. In addition, the higher error associated with DEMs derived from technical cartography (and thus from photogrammetric techniques that are not distinguishing between vegetated areas and bare soil) can not be taken into account if simple DEM differencing is carried out. This highlights the importance of assessing the uncertainty associated with the DoD maps by considering error propagation, before computing a quantitative mass balance of topographic change.

The magnitude and direction maps were used to outline the area, which was affected by the landslide. To quantify the individual DEMs’ error, “stable” areas were identified and analysed, retrieving the main statistics for these areas. This allowed the computation of the systematic error, as well as the standard deviation between pre- and post-event DEMs on a per-cell basis, which is a measure of the random error.

For a more accurate DoD analysis, the GCD 5 (Geomorphic Change Detection) extension for ArcGIS (Wheaton et al., 2010) was used. The software provides different methods to calibrate the DoD calculation, in order to take into account DEM uncertainty and error propagation into the DoD. One of the classical approaches is the “minimum level of detection” (_min_LoD), as introduced by Brasington, Rumsby & McVey (2000) and Fuller et al. (2003). This limit defines a threshold for the detection of elevation changes. Elevation changes that are larger than the _min_LoD are assumed true, whereas values below this limit are associated with the propagated error and discarded. For the present analysis, uniformly distributed _min_LoDs of 0.45 m and 1.10 m were considered for the LiDAR-based and “cartography” DoDs, respectively. These values were the maximum random errors retrieved for the “stable” areas.

The geometric properties of the landslide, including the failure area, its length and width in a map view, as well as the maximum failure depth, were measured from the resultant DoD maps. In addition, the mass balance of topographic change associated with the landslide, including the initial failure volume, was computed.

### Numerical modelling

For the 3D modelling the software DAN3D (Hungr & McDougall, 2009) was used. DAN3D is a meshless numerical method, adapted from smoothed particle hydrodynamics that uses an integrated two-dimensional Lagrangian solution and allows for the simulation of landslide runout across complex 3D terrain without mesh distortion problems. The software divides the rock mass into numerous particles that flow according to the underlying topography, based on a selected rheology.

Among the available 3D codes, DAN3D was selected because it allows the user to take entrainment of material during landslide runout into account and permits the selection of a maximum erosion depth along the runout path. The growth rate of the landslide volume due to material entrainment follows an empirical approach that considers the user-specified parameter E (erosion rate, (m^−1^)), which represents the increase in the volume of landslide material per unit of displacement, and must be adjusted by trial-and-error (McDougall & Hungr, 2005). The importance of material entrainment in case of the Le Laste landslide is emphasised by the entrainment ratio (Hungr & Evans, 2004), which exceeds 0.65.

The simulation of the landslide followed a trial-and-error back-analysis. The parameters that govern the basal resistance of the landslide were modified until the characteristics of the model (e.g., extend, depth of deposit, velocity) approximated those of the real event. The landslide characteristics were obtained from both the DoDs and seismic data (retrived from OGS, Istituto Nazionale di Oceanografia e Geofisica Sperimentale). Five seismic stations that are deployed around Mt. Antelao recorded the ground vibrations linked to the Le Laste landslide. The seismic records show a first peak, which is related to the detachment of the original rockslide from the summit of Mt. Antelao, and a succession of pronounced seismic signals about 10 to 15 s later. This strong second signal lasted around 30 s. It resulted from the impact of the material crashing onto the Antrimoia Valley, after the initial fall of 600 m. Therefore, the main part of the moving mass should have reached the Antrimoia Valley about 40 to 45 s after the initial failure. The simulated landslide velocity could be calibrated based on this information.

The DAN3D code requires three input files containing the following data on a fixed grid: (1) the elevation of the path along which the particles move, (2) the initial depth of the failure material, and (3) the material distribution along the path. Herein, the erosion map was used to distinguish between zones with (runout path beyond scar) and without (within failure scar and within the deposit area) significant material entrainment. Moreover, since DAN3D does not account for porosity variations due to fragmentation, the initial failure block was bulked by 25% at the source in order to consider volume increase due to material break up after failure.

To reduce the computational time of the simulation, and because the DAN3D code only allows 1,000 nods in *x*- and *y*-direction, the cells of the input files were resampled to a 5 m resolution. This enabled a direct comparison of the simulation results using (1) the integrated LiDAR and cartography derived pre-event DEM, and (2) the cartography derived pre-event DEM as input files. The availability of both pre- and post-event DEMs and the resultant DoD maps allowed an estimation of the uncertainties associated with the development of the different input data files.

## Results

### DoD analysis

**Figure 5:**
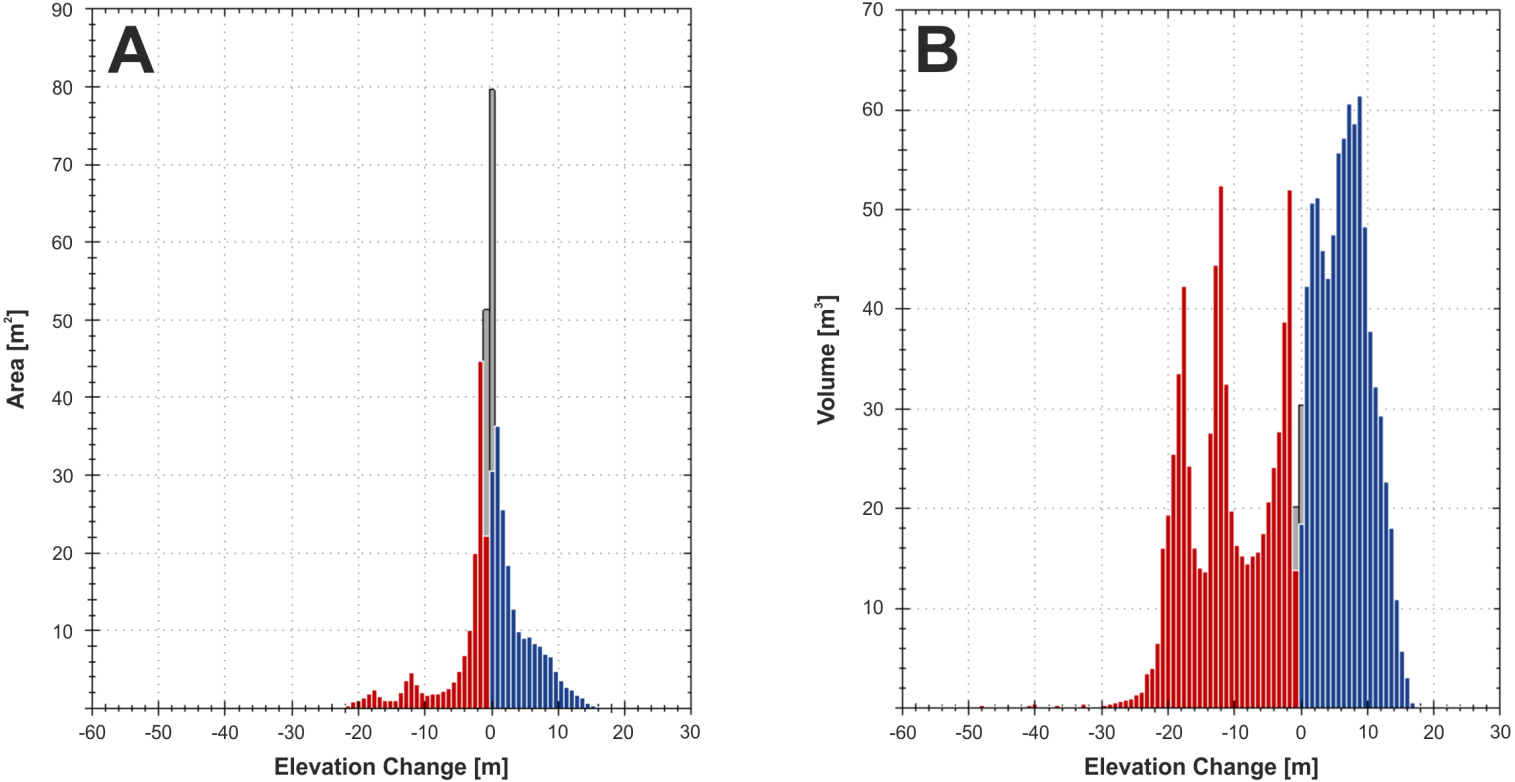
Elevation change distribution computed from integrated pre-event LiDAR and technical cartography and post-event LiDAR data. Areal (A) and volumetric (B) distributions of deposition (blue) and erosion (red). Values that were discarded during the DoD analysis are shown in grey.

**Figure 6:**
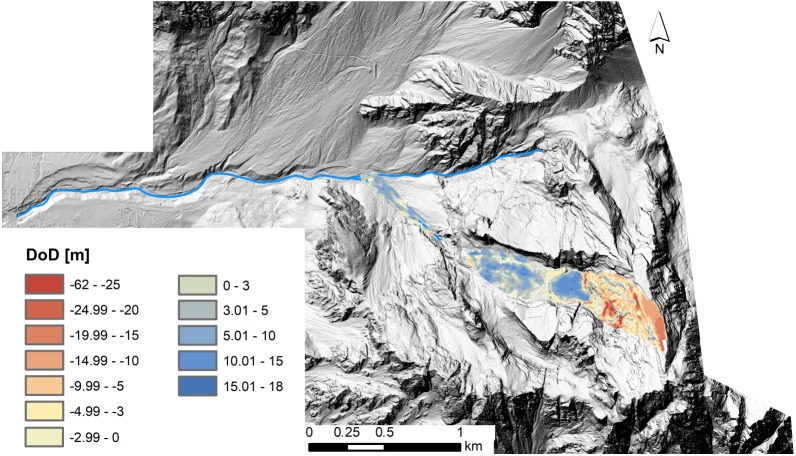
Volumetric and spatial distribution of geomorphic changes related to the Le Laste landslide. Erosion (red) and deposition (blue) patterns were computed from integrated pre-event LiDAR and technical cartography and post-event LiDAR data.

**Figure 7:**
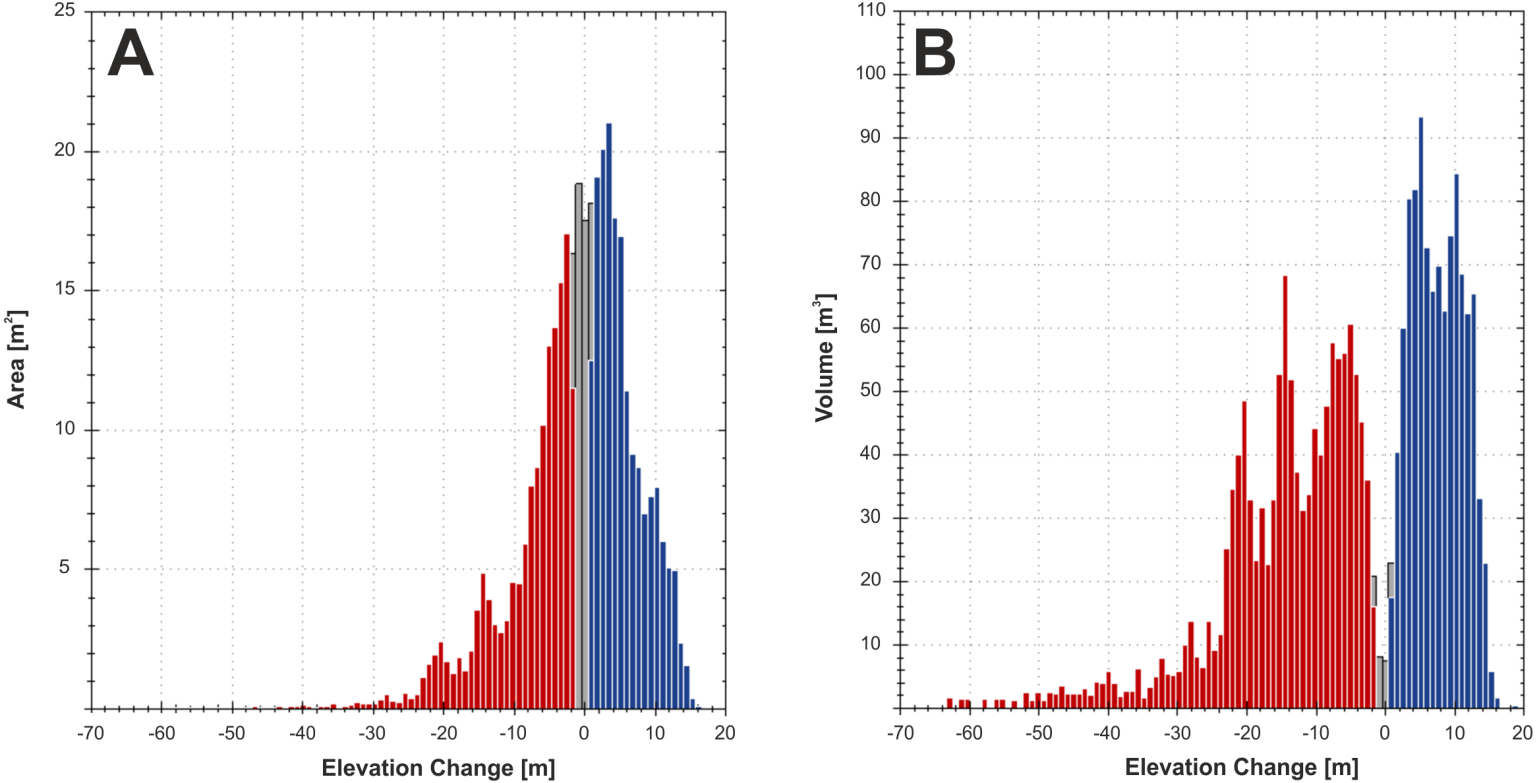
Elevation change distribution computed from 2007 pre-event technical cartography and 2015 post-event LiDAR data. Areal (A) and volumetric (V) distributions of deposition (blue) and erosion (red). Values that were discarded during the DoD analysis are shown in grey.

**Figure 8:**
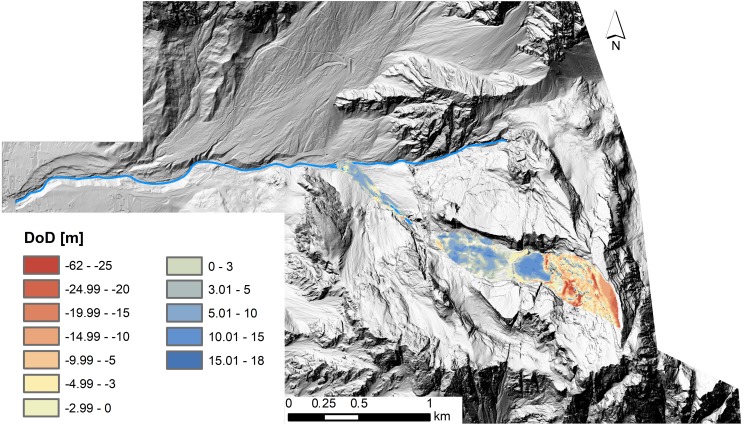
DEM of difference map elaborated from pre-event technical cartography and post-event LiDAR DEMs. In blue deposition and in red erosion.

Starting from the integrated LiDAR and pre-event cartography pre-event and LiDAR post-event DEMs of the Le Laste landslide, an elevation change distribution (Fig. 5) and a DoD map (Fig. 6) were computed and analysed in order to highlight erosion and deposition areas and to estimate their respective volume. A second DoD analysis was based on the 5 m pre-event DEM derived from technical cartography and the LiDAR-derived post-event DEM (Figs. 7 and 8). The main results of the two DoD analyses are listed in Tables 1 and 2.

**Table 1:** DoD analysis derived from pre- and post-event LiDAR datasets.

Attribute	Raw	Thresholded DoD estimate		
Areal				
Total area of erosion (m^2^)	179,294	150,156		
Total area of deposition (m^2^)	248,911	199,748		
Volumetric			±Error volume	% Error
Total volume of erosion (m^3^)	683,789	677,456	±67,570	10%
Total volume of deposition (m^3^)	813,820	801,952	±89,887	11%
Total volume of difference (m^3^)	1,497,608	1,479,406	±157,457	11%
Total net volume difference (m^3^)	130,031	124,496	±112,452	90%
Percentages (by volume)				
Percent erosion	46%			
Percent deposition	54%			
Percent imbalance (departure from equilibrium)	4%			

**Table 2:** DoD analysis derived from pre-event cartography and post-event LiDAR datasets.

Attribute	Raw	Thresholded DoD estimate		
Areal				
Total area of erosion (m^2^)	178,325	154,675		
Total area of deposition (m^2^)	202,800	179,650		
Volumetric			±Error volume	% Error
Total volume of erosion (m^3^)	1,268,862	1 256,094	±170,143	14%
Total volume of deposition (m^3^)	1,077,935	1,064,942	±197,615	19%
Total volume of difference (m^3^)	2 346 797	2 321 017	±367,758	16%
Total net volume difference (m^3^)	190 927	191 170	±260,768	−136%
Percentages (by volume)				
Percent erosion	54%			
Percent deposition	46%			
Percent imbalance (departure from equilibrium)	−4%			

In the first analysis, erosion and deposition volumes related to the landslide deviate by 4% from one another, which accounts for a net volume difference of 125,000 m^3^. However, it has to be noted that a high error of ±112,452 is associated with the total net volume. The original rockslide that detached from the summit of Mt. Antelao had an initial failure volume of 365,000 ± 13,463 m^3^. During its runout, the slide progressively transformed into a debris avalanche and entrained more material, increasing the total erosion volume to about 680,000 m^3^.

The travel path of the Le Laste landslide stretches over more than 2,000 m horizontally and about 1,600 m vertically. From the DoD map and aerial photographs (see Fig. 2), five discrete zones can be recognised. They are divided into: (1) The initial failure zone at the summit of Mt. Antelao, (2) a subvertical wall that connects the summit and the Antrimoia Valley, (3) the “Upper Deposit”, filling the Antrimoia Valley, (4) a rocky step, which separates the upper from the lower deposit, and (5) the “Lower Deposit”, which was accumulated along the Ru de Salvela.

The initial rockslide detached as one coherent block from Mt. Antelao. After sliding off the initial failure zone, between 2,800 and 3,100 m a.s.l., the block fell about 600 m down a 53° inclined slope onto the Antrimoia Valley. This fall caused the rockslide to break up completely and changed its runout behaviour into that of a debris avalanche. Before entering the Antrimoia Valley, an additional 255,000 m^3^ of material were entrained, while about 85,000 m^3^ were deposited along the path. The resultant debris avalanche that entered the Antrimoia Valley had a volume of 535,000 m^3^.

According to the DoD analysis, approximately 630,000 m^3^ of sediment accumulated within the Antrimoia Valley, forming the “Upper Deposit”. The debris avalanche filled a small depression right at the foot of the subvertical wall, before spreading out along the entire valley. This caused local erosion (35,000 m^3^) along the 32° inclined track, especially along the margins of the small depression in the upper part of the valley. After moving over the high gradient step which separates the valley from the Ru de Salvela, the material accumulated along the 18° inclined Ru de Salvela valley and formed the “Lower Deposit”. The “Lower Deposit” constitutes a mass of 80,000 m^3^, adding to the total deposit volume of over 800,000 m^3^. However, this data needs to be evaluated taking into consideration the debris flow that occurred on August 4, 2015. The debris flow initiated from the Antrimoia Valley and mobilised a volume of about 50,000 m^3^. Along its routing, it eroded parts of the deposit in the Ru Salvela creek and caused some fatalities downstream (Gregoretti et al. in press).

The comparison between the LiDAR-based DoD and “cartographic” DoD highlights a general overestimation of both erosion and deposition volumes in case of the “cartographic” DoD. A total erosion volume of 1,255,000 m^3^ and a total deposition volume of 1,065,000 m^3^ were computed with the “cartographic” DoD analysis. Although the imbalance between erosion and deposition is the same in both DoDs (i.e., ±4%), they have opposing signs. In the first analysis, the volume of the deposited material is higher than the volume of the eroded material. It is the other way around in the second analysis.

This could be linked to both, the errors related directly to the quality of the technical cartography and LiDAR data, and also to the fact that the scree slopes and the deposit areas in the Antrimoia Valley are subject to mass movements that may have likely occurred between 2007 and 2011.

### DAN3D modelling

To describe the runout dynamics of the Le Laste landslide a frictional rheology was selected for the numerical model during the calibration process:

\begin{eqnarray*}\tau =-{\sigma }_{\mathrm{z}} \left( 1-{\mathrm{r}}_{\mathrm{u}} \right) \tan \nolimits \phi =-{\sigma }_{\mathrm{z}}\tan \nolimits {\phi }_{\mathrm{b}} \end{eqnarray*}

where *τ* is the basal shear stress, *σ*_z_ is the total bed-normal stress, *r*_u_ = *u*∕*σ*_z_ with *r*_u_ the pore pressure ratio and u the pore fluid pressure, *ϕ* is the dynamic basal friction angle, and *ϕ*_b_ is the bulk basal friction angle (Hungr, 1995; Hungr & McDougall, 2009). The pore pressure ratio was assumed equal to 0, and an internal friction angle of 35° was used and not varied between runs. These values are considered appropriate for dry fractured rock (Hungr & McDougall, 2009), which compromised the bulk of the main mass. Both DoD maps (Figs. 6 and 7) showed that material was mainly entrained within the upper part of the landslide path. In the 3D model, volume changes due to material entrainment were accounted for by enabling erosion along the subvertical wall that connects the mountain summit and the Antrimoia Valley. An erosion rate of 0.00139 m^−1^ with a maximum erosion depth of 2 m was applied in the simulation that was based on the LiDAR-derived input files. For the “cartography” input files, an erosion rate of 0.00288 m^−1^ and a maximum erosion depth of 3.5 m were used. The higher values were chosen in order to factor the larger volume into the runout model. The remaining parameter *ϕ*_b_ was adjusted by matching the initial landslide velocity to the available seismic data.

The velocity of the initial failure could be reproduced with the set of numerical parameters suggested by McDougall (2006) and a basal friction angle of 24°. However, the code underestimated the runout of the debris avalanche and greatly overestimated the width of the “Lower Deposit”. In order to achieve a higher correlation between the DAN3D models and the DoD results, some of the numerical parameters were changed according to Sauthier et al. (2015). The smoothing length constant was set to 5, and the velocity smoothing coefficient and the stiffness coefficient were chosen as 0.02 and 200, respectively. Moreover, the number of particles (N) was increased to 3,000 to account for the large landslide volume.

**Figure 9:**
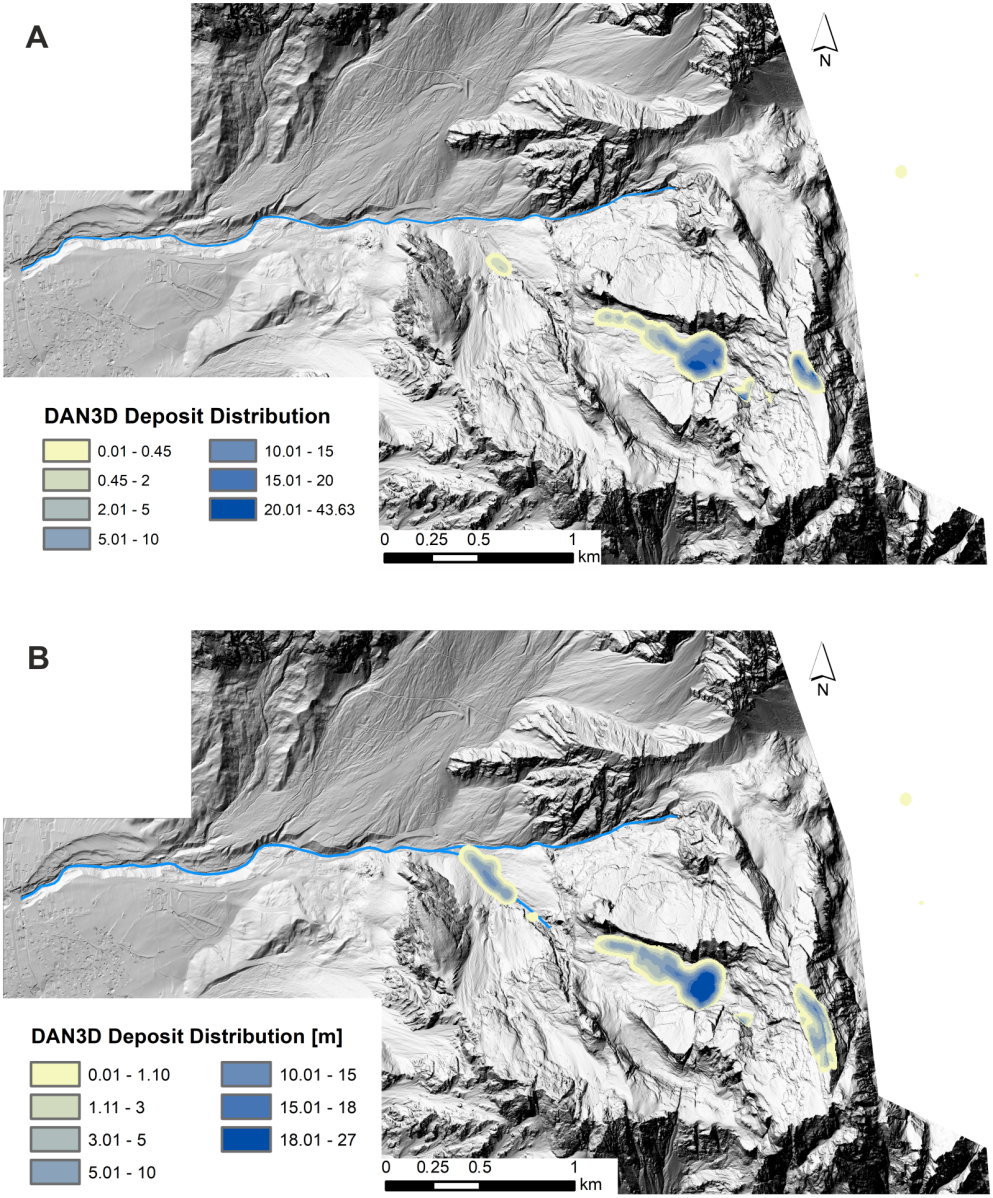
Maps of the numerical model results. (A) Pre and post-event LiDAR DEMs as input files (B) Pre-event technical cartography and post-event LiDAR DEMs as input files.

Figure 9 shows the simulated landslide and Tables 3 and 4 summarise the main results of the DAN3D simulations in comparison with the DoD analyses. Both, the initial failure volume and the total volume of deposited material are almost equal in the DoD analyses and their respective 3D models.

**Table 3:** Main results of DAN3D simulation based on LiDAR datasets.

Volume (m^3^)	DoD			DAN3D		Difference between DoD and DAN3D deposit	
	Erosion	Deposit	Balance	Erosion	Deposit		
Failure zone	367,266			459,665^a^	101,293		
Fall out					27,632		
Initial fall	255,133	86,625			27,905		
Mass entering Antrimoia Valley			535,774				
Upper deposit	36,207	630,605			613,098	−17,507	−2.8%
Lower deposit	14,749	78,582			6,294	−72 288	−92%
Total debris avalanche	677,456	801,952			776,223	−25,729	−3.2%

**Notes.**

a Initial thickness of failure material bulked with factor 1.25.

**Table 4:** Main results of DAN3D simulation based on pre-event cartography and post-event LiDAR DoD analysis.

Volume (m^3^)	DoD			DAN3D		Difference between DoD and DAN3D deposit	
	Erosion	Deposit	Balance	Erosion	Deposit		
Failure zone	324,082			406,582^*^	170,857		
Fall out					1,901		
Initial fall	853,702	102,203			9,668		
Mass entering Antrimoia Valley			1,075,581				
Upper deposit	64,980	813,286			831,757	−101,805	−2.3%
Lower deposit	13,330	149,453			142,621	−32,601	−4.6%
Total debris avalanche	1,256,094	1,064,942			1,109,940	−41,625	−4.2%

**Notes.**

a Initial thickness of failure material bulked with factor 1.25.

In the first model, the integrated pre-event LiDAR and technical cartography DEM was used to generate the relevant input files. The computed total volume of deposition deviates from the DoD result by only −3.2% (i.e., 25,000 m^3^). The simulated volume of the “Upper Deposit” was 615,000 m^3^, which accounts for an error of 17,500 m^3^. The “Lower Deposit”, on the other hand, was greatly underestimated by the dynamic model. According to the DoD analysis, about 80,000 m^3^ of material were accumulated along the Ru de Salvela. In the simulation, only a little over 6,000 m^3^ were deposited. This could be either a limitation of the numerical reconstruction or, due to a secondary phenomenon that mobilised material and transported it from the Upper to the Lower deposit. Furthermore, about 100,000 m^3^ of the material remained within the failure zone and more than 25,000 m^3^ fell off to the eastern side of Mt. Antelao. This material is, therefore, missing in the deposits.

The results of the “cartography” simulation also fit the respective DoD results quite well. Compared to the first simulation slightly more material remained within the failure zone (about 15% of the total deposition volume), but only about 2,000 m^3^ of material fell off the eastern side of Mt. Antelao. The volume of the “Upper Deposit” deviates by 2.3% from the volume derived from the DoD analysis and the simulated volume of the “Lower Deposit” also deviates by only 10,000 m^3^ from the DoD result.

In both models, the length and width of the “Upper Deposit” are simulated quite accurately. However, the numerical models greatly overestimated the maximal deposit thickness (up to 12 m), and the spatial distribution of deposit maxima and minima deviate from the DoD maps. The “Lower Deposit” is generally not very well represented in the LiDAR-based model. In the second simulation, the total length is underestimated and the foot of the material does not reach the Ru Secco.

## Discussion

### Co-registration between pre- and post-event DEM

The elevation difference between pre- and post-event DEMs is an essential element for the calculation of the accumulated volume and for the identification of erosion and deposition patterns. The results are extremely sensitive not only to errors from the DEMs’ derivation, but also to errors in their co-registration. Pre- and post-event DEMs may differ in various ways, including differences in the coordinate system, the positional accuracy, and other aspects (e.g., Chen et al., 2014).

Herein, we focused on “stable” areas and the proper alignment of the subvertical walls surrounding the Antrimoia Valley during co-registration. The maximum random errors retrieved from the “stable” areas were considered as _min_LoD for the DoD computation. The lower _min_LoD value (0.45 m) used in the LiDAR-derived DoD shows that the point cloud alignment was more accurate, mainly because of the higher resolution of this pre-event data. However, the errors along the sub-vertical walls remained high in both DoDs, even after co-registration of the pre-event DEMs. These errors can be largely attributed to the errors from the original LiDAR and technical cartography datasets.

Although LiDAR data offers high-resolution DEMs at the m-scale (Tarolli, Arrowsmith & Vivoni, 2009), the representation of the actual topography is a function of the density and spatial distribution of survey points (Brasington, Rumsby & McVey, 2000; Cavalli & Tarolli, 2011). Point density becomes especially important on steeply inclined slopes, where the horizontal error in the survey may introduce an “apparent” error in the elevation value (Hodgson & Bresnahan, 2004; Cavalli et al., 2017). Therefore, although the point density of the pre- and post-event LiDAR survey was high enough to compute 1 m DEMs, horizontal errors may have significantly increased vertical errors along sub-vertical walls. Moreover, because only the grids derived from LiDAR data and not the original point clouds were available, the accurate alignment of the pre- and post-event DEMs was even more difficult.

Technical cartography maps were used to generate a pre-event DEM because these maps are usually available and cover large areas of a country at different scales. However, they bear certain drawbacks, such as the loss of characteristic topographic features during the DEM generation (Heitzinger & Kager, 1998). Nevertheless, the comparatively easy access to these datasets makes the evaluation of their effectiveness in multi-temporal DEM computation extremely crucial.

Apart from the errors related to the original datasets, considering the time period between the pre- and post-event DEMs is crucial. As the area around Mt. Antelao is prone to debris flows and other landslides, it is very likely that the topography changed in between the different data acquisitions. For this reason, “stable” areas were selected to estimate the propagated error for the DoD analyses. However, it has to be noted that the pre- and post-event LiDAR surveys were conducted in November and October, respectively. By that time, the area around Mt. Antelao was already snow-covered. This snow cover also adds to the vertical error, as LiDAR pulses cannot penetrate it (Deems, Painter & Finnegan, 2013).

### Reconstruction of the Le Laste landslide

#### The landslide as described by the DoD

The results of the DoD analysis are in agreement with the observations derived from aerial photograph interpretation in Fig. 2, and field survey (Baglioni & De Marco, 2015). Five discrete zones could be identified, and an initial failure volume of 365,000 m^3^ was computed, which exceeds the previous estimate of 300,000 m^3^.

Tables 1 and 2 show that the volume of total erosion and the accumulated material is roughly balanced in both DoD analyses. However, while the volumes in the LiDAR-derived DoD deviate positively from the equilibrium, in the “cartography” DoD the total volume of erosion is larger than the total deposit volume.

In the LiDAR-based DoD analysis, the deposit volume exceeds the volume of eroded material by 125,000 m^3^. The imbalance between erosion and deposition decreases, if fragmentation of the initial rockslide is considered. The fragmentation causes the volume of the original rockslide to increase, thereby increasing the total erosion volume to 770,000 m^3^ (bulking of the initial failure block by 25%). This, in turn, reduces the departure from equilibrium to 2.2%. The difference between the expected and the actual deposit volume can also be related to the time lag between the occurrence of the Le Laste landslide in 2014 and the LiDAR data acquisition in 2015. Within this time period, the topographic surface was altered by sediment transport processes such as debris flows and human activity. Already right after the landslide had occurred, rainfall (Fig. 3) caused more material to be eroded and enabled the advancement of the accumulation front. Moreover, parts of the material of the Le Laste deposit in the Antrimoia Valley and Salvela Creek were remobilised during the August 4, 2015 debris flow, described in Gregoretti et al. (in press), which benefited from LiDAR data analysis carried out by Boreggio, Bernard & Gregoretti (in press).

Lastly, it has to be noted that the large errors in the DoD maps, which are recognised along the steep slopes of Mt. Antelao, can affect the volumetric estimations of erosion and deposition. Along these subvertical walls, erosion rates of over 50 m are reported. Considering that the maximum thickness of the original rockslide did not exceed about 25 m, it is rather unlikely for the erosion depth along the runout path to be larger.

Comparing the two DoD maps (Figs. 6 and 8) shows that these errors are smaller in the LiDAR-derived DoD. Errors would have been even lower if 2011 LiDAR data were available for the upper part of the catchment too. This highlights the importance of meter- to sub-meter-scaled topographic data for the investigation of steeply inclined slopes. Owing to the finer resolution of the pre-event DEM, the error could be minimised through co-registration. This ensured that the elevation change distribution within the landslide area was not strongly affected by these errors.

Regarding the comparison between the LiDAR and technical cartography analyses, it can be argued that the latter approach may relate to a systematic magnitude overestimation. Nevertheless, it allowed the correct computation of erosion and deposition patterns, and the correct estimation of volume difference magnitudes. The approach, while lacking in accuracy, can be considered a precautionary reference scenario, and might be a valuable option when high-resolution datasets are not available.

#### Comparison of DoD analysis and DAN3D simulation

Multi-temporal DEMs have proved to be a useful tool to enhance the conformity between numerical models and the real event that they simulate (Bossi et al., 2015). However, the reliability of the model is strongly dependent on the accuracy of the input files, which are obtained from the DoD analyses. These files can contain quite large source errors. Therefore, the numerical model was calibrated with seismic data to ensure its reliability and its applicability for further risk studies. Seismic records of a landslide, in fact, provide velocity information, which cannot be obtained from DoD data. With this additional information, the simulated landslide velocity can be verified and the model’s accuracy is enhanced.

The simulated landslide velocity conformed to the velocity information obtained from the seismic data, which showed that the main part of the initial rockslide fell onto the Antrimoia Valley after about 40 s. In addition, the volume and shape of the “Upper Deposit” in both models show a good correspondence with the DoD maps. Nevertheless, there are some notable discrepancies between the landslide models and the DoD maps, especially in the representation of the “Lower Deposit”.

The post-event data, which is used in the DoD analyses, was acquired 11 months after the landslide had occurred. Therefore, the topographic changes illustrated in the DoD maps do include geomorphic changes that occurred in this period and which are not directly linked to the initial landslide. To account for these potential post-event processes, the DoD analyses will not be referred to as “real event” in the following comparison with the dynamic runout models.

It was shown that the depth of the “Upper” deposit was greatly overestimated in both 3D models. However, the total volume of the simulated deposit differs only 2–3% from the DoD maps. It is likely that erosion and deposition processes within the deposit caused a redistribution of the accumulated material in the months after the landslide, causing changes in deposit thickness, as well as in the spatial distribution of deposit maxima and minima. Especially the simulated sediment accumulation, filling the small depression at the foot of the sub-vertical wall connecting the summit of Mt. Antelao and the Antrimoia Valley, is likely to fail during heavy rainfall or snowmelt. This part of the “Upper” deposit has the greatest thickness and forms sediment mounds with slope gradients >25°. However, the deposit’s potential redistribution is not considered in the simulations, but must be taken into account when evaluating the DoD results.

The DAN3D code also underestimated the length and overestimated the width of the “Lower” deposit in the cartography-based simulation, while its volume was generally underestimated in the LiDAR-based model. The difference in deposit width in the “cartography” simulation can be largely attributed to the application of a _min_LoD of 1.10 m in the DoD analysis. Values beneath this threshold were discarded, which might cause an apparent reduction in the deposit width. The simulated deposit width does include these fine sediments, which increases the total deposit width.

The underestimation of the length or entire volume of the “Lower” deposit in the cartography- and LiDAR-based analysis can again be related to the August 4, 2015 debris flow (Gregoretti et al., in press). The debris-flow event remobilised the Le Laste deposit moving and redistributing the material downstream, thus changing the local deposit.

Another problem was the volume (about 100,000 m^3^ and 170,000 m^3^ for the first and second simulation, respectively) that remained within the failure zone and therefore, is missing in the landslide deposits. This could be attributed to the intrinsic quality of the technical cartography-derived pre-event DEM, which was used to cover the failure area in the input files of both simulations. Uncertainties in the DEM could affect the topographic representation in the model, causing changes in the runout behaviour of the material. In addition, the DAN3D software discretises a landslide mass into numerous particles that move according to the underlying topography, based on a selected rheology (Hungr & McDougall, 2009). Thus, instead of one massive initial sliding block, the rock mass was represented by numerous individual particles. This resulted in the lower particles to remain in place, as the slope angle was not great enough for them to overcome their critical angle of friction. However, in reality, the whole block slid according to the underlying stratigraphic layering, allowing all of the material to fall onto the Antrimoia Valley. The absence of this precondition explains parts of the material remaining in the failure zone. Finally, it has to be noted that bulking of the initial failure block by 25% in the simulation, caused changes in the initial block’s thickness. The bulking is implemented by increasing the block’s thickness to encompass the increase in volume due to fragmentation. Therefore, parts of the block locally exceed the surrounding material, which can cause an abnormal behaviour (i.e., material remaining within the failure zone, as particles remain on top of the surrounding rock).

### Landslide risk at Mt. Antelao

In the past, the region around Mt. Antelao was subject to numerous devastating mass movements (Panizza et al., 1996; Pasuto & Soldati, 1999; Pasuto & Soldati, 2004), and in recent years, landslide risk has been increasing significantly. Accumulated loose material from the Le Laste landslide along the Antrimoia and Ru de Salvela valleys has the potential to give rise to large debris flows during heavy rainfalls (Gregoretti et al., in press). These threaten the downstream community not only directly, but also through their potential to dam rivers (Fig. 2C). This debris flow hazard becomes even more important considering the increase in tourism and tourist development linked to the Alpine Ski World Championship in 2021. Therefore, the correct delineation and quantification of sediments produced by the Le Laste landslide from DoD analyses are crucial for debris flow forecasting and related hazard assessment.

Moreover, the soil parameter values obtained from this study may have the potential to act as input parameters for further hazard assessments in the region. The simulation of potential landslide runout enables a more profound development of hazard maps, which can be used to set up structural mitigation measures and evacuation plans. The recognition of these changes in risk conditions and the promotion of risk awareness within the community is of utmost importance for the implementation of effective mitigation strategies.

## Conclusion

This paper investigated the potential of multi-temporal DEM analysis, with DEMs from different sources and at different resolutions, for geomorphic change detection. It was shown that the accuracy of the analysis strongly depends on the original accuracy of the individual DEMs. The comparison between the DoD analysis based on LiDAR and cartographic data showed that the latter approach may be suitable for volumetric estimations of erosion and deposition related to large landslides, if high-resolution datasets are not available. The observed systematic magnitude overestimation can be considered a precautionary reference scenario.

The combination of the DoD results with seismic records of the Le Laste landslide proved to be very useful for the accurate calibration of the 3D dynamic model. The availability of velocity data from the monitoring system, allowed for a more reliable selection of soil parameters in the landslide simulation. This underlines the importance of seismic network setups in landslide-prone areas, not only for forecasting, but also for a more effective investigation of landslide dynamics.

Landslide hazard assessment and mitigation will become increasingly important in the area of Mt. Antelao. Debris-flow hazard, linked to the landslide-induced sediment deposits, and the rising number of tourists, infrastructure development and human activity, results in an increased landslide risk. Therefore, a good description of previous landslides in the area, like the Le Laste landslide, is crucial for the implementation of mitigation strategies. In addition, a reliable numerical model enables the calibration of soil parameters, which can be used to simulate the runout of possible future rock slides.

##  Supplemental Information

10.7717/peerj.5903/supp-1 Supplemental Information 1 Input grids (source file, erosion map and surface) used for the DAN3D analysis DAN3D requires input files in .grd format. Here the files have been converted into TXT since it is an open format. Path Topography file: matrix defining the topography on which the flow occurs. Source Topography file: matrix defining the vertical depth of the detaching mass at the initial time. Erosion Map file: matrix defining the areas (4 in our case) where different properties are assigned to the flow mass and to the soil bed (maximum erosion depth)

## Additional Information and Declarations

### Data Availability

The following information was supplied regarding data availability: The raw data are provided in Supplemental File.
